# Augmented Reality–Enhanced Simulation in Surgical Education: A Scoping Review of Implementation, Skill Retention, and Equity in Low-Resource Settings

**DOI:** 10.7759/cureus.102079

**Published:** 2026-01-22

**Authors:** Ee Hng Ian Lim, Li-Zhang Tan, Hui Min Foo, Samuel S Adrian

**Affiliations:** 1 Medicine, General Surgery, Cardiff University, Cardiff, GBR

**Keywords:** augmented reality (ar), augmented reality surgical navigation, education equity, low resource setting, medical education and training, medical education technology, skill retention, skills and simulation training, surgical education, surgical skills-based training

## Abstract

Many developments have been made to enhance surgical training, including the usage of augmented reality (AR) simulation. However, its implementation within surgical training programs, impact on skill retention, and applicability in low-resource settings remain unclear. A scoping review was used to explore the role of AR technology and its implementation in surgical education, maintenance of skill retention, and equity and access in low-resource settings. A systematic search was conducted across four databases, which yielded 185 records, of which 30 met the inclusion criteria. Most studies were published after 2019 and evaluated AR interventions in simulated environments involving medical students or residents. AR intervention was primarily used for visual guidance, telestration and image overlay. Evidence for skill retention remains limited, with only four studies assessing retention outcomes. Short-term improvements in task performance and procedural planning were reported, while skills transfer to clinical environments and long-term retention remain underexplored. Several studies created low-resource, reusable AR systems, reporting increased learner confidence and procedural knowledge; however, these studies were limited by small sample sizes and a reliance on subjective outcome measures. In conclusion, current evidence suggests that AR has the potential to become a useful tool for surgical education. However, the literature remains preliminary, with more longitudinal, objective and equity-focused studies required.

## Introduction and background

Surgical training increasingly faces challenges like limited operative exposure, patient safety concerns and disparities in access between low and high-income areas [1]. Simulation-based technology has emerged as a solution to address these issues, enabling autonomous and low-stakes surgical training [2]. Among upcoming simulation technologies, augmented reality (AR) provides a combination of digital imaging with physical interactions, making it a useful adjunct for traditional surgical teaching [3].

AR is defined as technology that overlays computer-generated digital content onto the real-world environment in real time [4]. This differs from virtual reality (VR), which consists entirely of virtual objects, and mixed reality (MR), that merge real and virtual worlds such that physical and digital objects coexist and are presented together [4]. By augmenting digital media over the physical environment, AR can serve as a technological platform to deliver surgical education, such as displaying surgical techniques over a human cadaver [5]. Despite growing interest and technological advances, it is unclear whether AR is effective in its implementation in surgical education, including its usefulness in skill retention and applicability in low-resource settings. 

This scoping review was used to provide a comprehensive overview of the research conducted within AR in surgical education, as well as identify potential gaps in the literature. Specifically, this review was conducted to evaluate the approaches to integration of AR in its: (1) implementation in surgical education, (2) skill retention, and (3) equity and feasibility in low-resource settings.

## Review

Methods

Study Design

The protocol for this review was conducted using the guidelines of the Preferred Reporting Items for Systematic Reviews and Meta-Analyses (PRISMA), specifically the PRISMA extension for scoping reviews (PRISMA-ScR) [6]. Institutional Review Board (IRB) and ethical approvals were not required, as this was a secondary data synthesis of published literature. Moreover, the personal information of participants was not revealed in the reviewed studies, and no external funding was involved. 

Inclusion and Exclusion Criteria

To map out our objectives, this review follows the PICO (population, intervention, comparator, outcome) framework. The target population was medical students, doctors, and surgical trainees. The intervention investigated was an AR-based simulation. As a comparison, the population subjects had to use or have access to AR technology, versus a control setting (e.g., video tutorials). Some single-arm studies were also included to map the breadth of existing evidence and implementation experiences. The outcomes assessed included its effectiveness in surgical education, long-term skill retention, or equity and feasibility in resource-limited settings. In addition, peer-reviewed articles were included if they were: in English, primary research, and from any publication year. 

**Figure 1:**
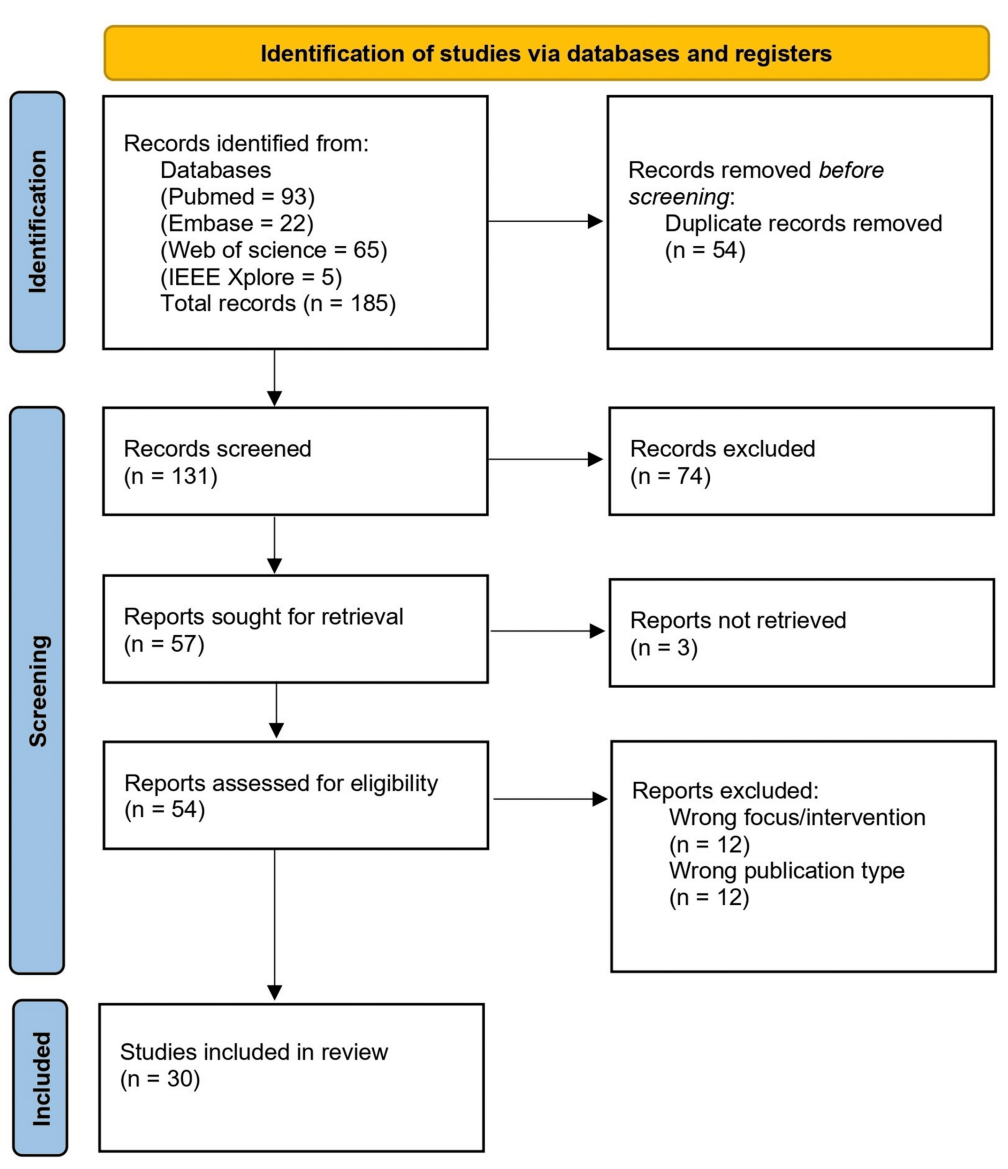
PRISMA flow chart PRISMA: Preferred Reporting Items for Systematic Reviews and Meta-Analyses

To be included in the review, studies needed to focus on AR-based simulations in surgical training, adhering to the PICO framework. Examples of why papers were excluded involved VR simulations, not written in English, first-aid/multiple casualty incident simulations, review-type articles and animal studies without human training outcomes. Reports where full text was not retrievable (e.g., abstract-only publications) were not assessed for eligibility and excluded under “reports not retrieved” as shown in the PRISMA-ScR flow diagram (Figure 1).

Search Strategy and Planning

To identify the relevant studies, the following bibliographic databases were searched on the 21st of August 2025: Embase, Web of science, IEEE web Xplore and Pubmed. Boolean logic, keywords, phrases and synonyms were applied to title and abstract field to expand the literature search. Controlled vocabulary (e.g., MeSH) was not used because emerging immersive technologies are frequently described interchangeably (e.g., AR, MR, and VR) without consistent or formal definitions, resulting in unreliable indexing. An example of the search strategy is included in the Appendices section. The final search results were uploaded to Rayyan (Rayyan Systems Inc., Cambridge, MA, US), a web tool for sorting articles. Rayyan was then used to detect duplicate studies which were subsequently removed. Three researchers (IL, EF, LZT) individually sorted abstracts of articles based on the inclusion and exclusion criteria. An external reviewer (SS) was used to resolve any conflicts that arose during decision-making. Next, the abstracts that were included were reuploaded onto Rayyan as full-text articles. The three researchers then evaluated the full text of the included studies. Further conflicts between the reviewers were resolved in a 2:1 favor. A review of the finalized studies was conducted by all the researchers to appraise their quality and check for adherence to the eligibility criteria.

Results

The literature search identified 185 records. After removing 54 duplicates, 131 records were screened by title and abstract. During this process, 74 results were excluded. 57 full-text articles were sought for retrieval, and three were removed due to lack of full-text availability. The remaining 54 articles were assessed for eligibility, with 30 studies included in the final synthesis. 

The articles are described in Table 1 together with the publication year, country, study design, learner level, surgical specialty and AR intervention. 

**Table 1:** Summary of the included articles USA: United States of America; UK: United Kingdom; RCT: randomised control trial; AR: augmented reality.

Study (First author)	Year	Country	Study design	Learner level	Surgical specialty	AR intervention
Vera et al. [7]	2014	USA	RCT	Medical students	Laparoscopic surgery	AR telementoring overlays guiding suturing task
Heinrich et al. [8]	2021	Germany	Pre-clinical within-subject crossover trial	Residents, consultants	General surgery	HoloLens-based AR pointer providing task guidance
Ghenbot et al. [9]	2024	USA	Cadaveric within-subject experimental study	Medical student, resident, consultants	Neurosurgery	HoloLens 2 AR navigation with spine anatomy overlays
Acar et al. [10]	2024	USA	Within-subject experimental study	Residents/Fellows, consultants	Urology	Mixed-reality gaze-sharing AR for ureteroscopy training
Kambakamba et al. [11]	2024	Switzerland	Prospective pilot study	Senior residents	General surgery	Smart-glasses AR telementoring during procedures
Lovett et al. [12]	2024	USA	RCT	Medical students	General surgery	HoloLens AR suture-guidance overlays
Yeo et al. [13]	2011	Canada	RCT	Residents, medical/bio-engineering students	Interventional radiology/spinal surgery	AR-guided needle placement using hybrid simulator
Felinska et al. [14]	2023	Germany	Randomised-controlled crossover trial	Medical students	Laparoscopic surgery	iSurgeon (AR telestration) Visual guidance during training.
Nagayo et al. [15]	2022	Japan	RCT	Medical students	General surgery	HoloLens 2-based AR training providing task guidance
Guerrini et al. [16]	2024	Italy	Prospective observational study	Medical students	Neurosurgery	UpSurgeOn simulators (Mycro & AR neuroanatomy)- microsurgical suturing and AR neuroanatomical models.
Cizmic et al. [17]	2023	Germany	Randomized controlled two-arm study	Medical students	Laparoscopic surgery	iSurgeon (AR telestration) visual guidance during training.
Feifer et al. [18]	2008	Canada	Prospective cohort study	Residents	Urology	ProMIS hybrid simulator (Haptica) –provides haptic feedback and records movement efficiency.
Logishetty et al. [19]	2019	UK	RCT	Medical students	Orthopaedics	HoloLens headset with MicronTracker – Provides real-time holographic feedback during simulated THA.
Alaraj et al. [20]	2013	USA	Descriptive early validation study	Residents	Neurosurgery	ImmersiveTouch system – AR surgical simulator for neurosurgical procedures
Wu et al. [21]	2025	Switzerland	Development/feasibility study	Residents	Orthopaedics	Custom HoloLens 2 AR-based surgical simulator
Berger et al. [22]	2025	Austria	Prospective cohort study	Residents	Neurosurgery	Video + AR training with neurosurgical simulators
Coelho et al. [23]	2020	Brazil	Phase I mixed-methods validation study	Consultants	Neurosurgery	Mixed-reality neurosurgical simulation platform
Alonso-Silverio et al. [24]	2018	Mexico	Experimental development and validation study	Consultants, residents, medical students	General surgery	Low-cost hybrid laparoscopic trainer with AR guidance
Moglia et al. [25]	2024	Italy	Preclinical usability and performance study	Consultants, residents, medical students	Orthopaedics	Holoknee (AI + MR) – AR knee planning and guidance with AI 3D segmentation.
Barzilai et al. [26]	2025	Israel	RCT	Medical students	Otolaryngology	HoloLens 2 - AR-guided mastoidectomy simulator
Wolf et al. [27]	2024	USA	Experimental	Surgical trainees	General surgery	Tablet-based AR surgical training environment
Shaaban et al. [28]	2024	USA	Cross-sectional pre/post survey study	Medical students, residents	Neurosurgery	Hybrid physical–AR anatomical simulation model (UpSurgeOn system)
Cuba et al. [29]	2024	Switzerland	Prospective performance and learning curve study	Residents	Neurosurgery	Patient-specific mixed-reality surgical simulator (HoloLens 2)
Ropelato et al. [30]	2020	Switzerland	Experimental validation study	Physician trainees	Opthalmology	Custom AR microsurgery training simulator with HoloLens display
P. Jain et al. [31]	2024	India	Two-phase mixed-methods user perception study	Residents, consultants	Laparascopic surgery	Conceptual AR simulator for surgical education
Shepard et al. [32]	2025	USA	Prospective randomized crossover trial	Residents	Neurosurgery	Remote mixed-reality training using Vuzix smart glasses
Aydoseli et al. [33]	2024	Turkey	Quantitative descriptive survey study	Residents	Neurosurgery	Illumetry XR screen with ArSurgeon software used to display segmented 3D MRI and CT images of neurosurgical cases
Nugent et al. [34]	2013	Ireland	Prospective validation and skill acquisition study	Consultants, Medical students, surgical trainees	General surgery	ProMIS AR laparoscopic simulation system
Stone et al. [35]	2022	USA/Netherlands	Proof-of-concept and beta testing study	Surgical trainees/ residents	Urology	Custom AR headset with heads-up display for task guidance
Colman et al. [36]	2025	UK	Mixed-methods multicenter pilot validation study	Trainees, consultants	General Surgery	LapAR™ augmented-reality laparoscopic simulator

Most included studies were published after 2019. Early articles focused on hybrid simulators and guidance using AR, whereas more recent studies emphasize head-mounted displays, mixed reality simulators, AI integration and telementoring. Residents dominated feasibility, validation, and learning-curve studies, while medical students were the primary participants of randomized control trials (RCTs), particularly in general surgery and laparoscopic training (Table 1). 

Implementation of AR-Enhanced Simulation

Across included studies, AR technology served three primary roles: (1) visual guidance/image overlay, (2) navigation and anatomy visualisation, and (3) telestration and gaze-sharing. Visual guidance and image overlay was mainly applied to RCTs, involving novice learners such as medical students (Table 1). This role primarily evaluated short-term performance outcomes and had the largest representation among study types. In contrast, navigation and anatomy visualization was often applied to specialized surgical fields, such as ear, nose, or throat (ENT) or neurosurgery, with outcomes focusing on enhancing spatial understanding and pre-operative planning (Table 1). Telestration and gaze-sharing were examined in fewer studies and were mainly implemented with senior trainees, emphasizing real-time expert feedback and mentorship (Table 1). 

Reporting of study settings and curricular integration was variable. Twenty-two out of the 30 included studies explicitly reported study settings (73%), such as simulation/skills labs, operating theatres and home-based/remote training. Formal integration into surgical curricula was reported in six out of 30 studies (20%).

*Skill Retention Outcomes*

Skill retention is defined as the maintenance of acquired skills following a period without practice [37]. As seen from Table 2, four out of 30 studies assessed skill retention outcomes. 

**Table 2:** Summary of studies assessing skill retention MR: mixed reality; MCQ: multiple-choice questions; IP: in-person

Study	Retention interval	Retention type	Retention measure	Retention findings
Berger et al. [22]	14 days	Short-term	Tremor metrics, performance trend: reduction in wrist tremor over training.	Significant tremor reduction across groups and experience levels
Moglia et al. [25]	1 week between trials	Short-term	Task completion time, usability	Significant improvement in task speed, MR effective for training, planning and guidance
Wolf et al. [27]	1 week between AR training and cadaveric assessment	Short-term	Procedural knowledge MCQ, tablet-based procedural skills, cadaveric procedural performance	Significant improvement in tablet procedure knowledge and skills. No transfer to cadaveric skill performance
Shepard et al. [32]	2 months	Long-term	Procedural success, puncture attempts, procedure time, retention scores	Skills maintained at 2 months, MR training equivalent to IP training, preference of IP training due to hardware discomfort

Short-term retention was evaluated by three of the four studies [22,25,27]. The retention intervals ranged from one week to two months. Berger et al. reported a significant reduction in wrist tremor (56-69%) even after a one-week delay, consistent among junior and senior residents [22]. There were also improvements in task time completion after a one-week interval in Moglia et al., indicating short-term maintenance in procedural efficiency [25]. Wolf et al. also displayed improvements in AR procedural skills and knowledge (p<0.001) on tablet-based platforms. However, no significant improvement in cadaveric task performance was observed [27].

Long-term retention was assessed by one out of the four studies. With a two-month retention interval, urological residents were still able to carry out ultrasound-guided percutaneous nephrolithotomy in both AR and in-person training groups. Post-test to retention test yielded insignificant decreases in average performance scores. Procedural skill was maintained two months post-training [32].

Equity and Low-Resource Settings

Several studies (n=11/30) designed affordable, usable, and scalable simulation tools for surgical training in low-resource settings/low-middle income countries (LMICs) by using open-source approaches and local production. Many of these systems were created to reduce reliance on cadavers or high-cost infrastructure (Table 3).

**Table 3:** Summary of studies addressing equity and low-resource settings LMIC: low-middle income countries; AR: augmented reality; MIS: minimally invasive surgery; MR: mixed reality; XR: extended reality; OR: operating room.

Study	Equity focus/Objective	Key findings on equity	Challenges/Barriers	Reviewer notes
Guerrini et al. [16]	Address neurosurgical training gaps through sustainable, low-cost simulation programs targeting low-resource settings	Simulation increased interest in surgical careers and self-reported confidence; program model supports repeated training in low-resource regions	No control group; self-reported outcomes; short-term exposure; no skill or retention measures	Direct equity focus via Mission:Brain’s LMIC-oriented training and sustainability model
Coelho et al. [23]	Develop a reusable, lower-cost ventriculostomy simulator suitable for resource-limited training environments	Simulator demonstrated high perceived realism and usability; reusability and local production support affordability in LMIC contexts	No objective performance or retention outcomes; subjective measures only. Small expert-only sample.	Direct equity relevance; developed in an LMIC with explicit low-resource training rationale
Alonso-Silverio et al. [24]	Develop a low-cost, open-source AR training system for use in resource-limited settings	Significant task improvement with AR system. Demonstrated reliable, affordable, and objective laparoscopic skill assessment	No long-term retention or clinical transfer assessment. Small sample size.	Strong direct equity relevance; open-source, LMIC-developed, highly scalable
Barzilai et al. [26]	Evaluate AR-guided mastoidectomy using low-cost 3D-printed models as an alternative to cadaveric training	AR significantly improved novice performance; low-cost printed models support repeatable training	High cost of AR headset; single-session design; no LMIC deployment	Direct but moderate equity relevance; affordability discussed but tested in high-resource setting
Wolf et al. [27]	Tablet-based AR designed for distributed, portable training in austere/limited-access settings	AR improved knowledge and procedural planning; portable tablet allows deployment in field-like environments	No haptic feedback; short-term evaluation; no actual skill transfer to cadaveric performance	Directed toward access in resource-limited/expeditionary contexts; moderate equity relevance
Shaaban et al. [28]	Demonstrate AR-physical hybrid simulation as cost-effective, reusable, and scalable for low-cadaver-access / LMIC settings	Participants reported improved confidence and satisfaction; course setup eliminates biohazard and storage barriers, supporting scalable training	Single-session survey; no objective skill or retention data; conference setting limits realism	Direct high-equity relevance; explicit LMIC applicability and low-resource design
Jain et al. [31]	Develop cost-effective, scalable video-based AR workflow for laparoscopic/MIS training in low-resource settings	Strong user interest and perceived usefulness; scalable, low-cost workflow suitable for institutions without expensive hardware	Perception-only study; no hands-on performance or retention data; small follow-up cohort	High equity relevance; LMIC-focused, feasibility demonstrated; next step: prototype testing
Shepard et al. [32]	Evaluate remote MR training as an alternative to in-person instruction, potentially expanding access to smaller or resource-limited centers	MR training equivalent to in-person for skill acquisition and retention; enables remote expert guidance	Hardware cost and connectivity; small sample; single institution; trainee preference for in-person	Moderate equity relevance; demonstrates remote access potential but limited by equipment and connectivity
Aydoseli et al. [33]	Evaluate low-cost XR/AR neuronavigation for neurosurgery training, with potential applicability in low-resource settings	AR improved motivation and perceived anatomical mastery; device is affordable and easily reproducible	Single-session, subjective survey; no objective performance or retention metrics; not tested in LMICs	Moderate equity relevance; cost-effective design with potential for low-resource deployment
Stone et al. [35]	Evaluate remote AR telementoring platform to expand access and reduce travel/costs for surgical training	Remote AR enabled successful simulated procedures across geographic distances; inexpensive phantoms and consumer hardware support scalable training	Small sample, no objective skill metrics or retention testing; single-session; high-resource sites	Moderate equity relevance; strong feasibility demonstration with potential for low-resource deployment
Colman et al. [36]	Evaluate home-based AR laparoscopic simulator to increase accessibility and equity in early surgical training	Trainees improved completion time (52%), instrument path (38%), and qualitative feedback strongly positive; remote, low-infrastructure design supports broader access	Small sample, no real-OR transfer, no long-term retention; minor technical issues	High equity relevance; decentralized, low-resource-compatible AR training with strong feasibility and trainee acceptance

Across studies, AR interventions were associated with increased learner confidence, motivation, and procedural understanding, alongside improved task performance and procedural planning (Table 3). Several AR systems supported remote training, enabling repeated practice at low cost (Table 3). 

This scoping review synthesizes evidence in three areas: implementation of AR into surgical education, skill retention using AR, and equity and access in low-resource settings. Most AR systems were mainly focused on visual guidance and image overlay, primarily involving medical students or novice trainees (Table 1). Evidence on long-term skill retention, clinical transfer, and implementation in low-resource settings remains limited.

Discussion

Implementation of AR

Most AR interventions are carried out in simulated environments, limiting evidence on skills transfer to operating theatres, reflecting on the early developmental stage of AR surgical education [38]. The heterogeneity of AR systems presents a challenge to standardized curriculum integration, with variable performance metrics, training requirements, and inconsistent equivalence to in-person instruction [39]. AR provides a low-stakes environment for surgical training, potentially increasing competence, confidence, and patient safety [40]. Certain AR devices support remote or home-based practice, increasing training opportunities outside clinical settings [36]. However, the inconsistent reporting of study settings and limited documentation of curricular integration restricts assessments on the feasibility and scalability of AR training. Overall, AR provides a promising platform for surgical training, but effectiveness in clinical settings with standardization of performance metrics need to be evaluated. 

Impact on Skill Retention

The evidence on AR skill retention remains limited. Short-term retention outcomes suggest improvements in procedural efficiency (Table 2). However, only one out of the 30 studies assessed long-term skill retention, hence evidence for sustained skill transfer remains limited. Moreover, AR-enhanced training remained unable to transfer to cadaveric performance - indicating further refinement is needed before application to real-world contexts [26]. AR systems provide objective feedback on metrics such as skill scores, movement efficiency and technique consistency, allowing potential standardization of training compared with traditional hands-on sessions [38]. This finding, along with opportunities for autonomous practice and spatial guidance, may support skill retention; however, the extent to which these features confer long-term advantages over conventional methods remain uncertain and require further evaluation [41].

Equity and Access in Low-Resource Settings

AR-based simulations are frequently shown as tools to reduce disparities in surgical training by offering low-cost, reusable, and decentralized training opportunities [42]. However, most AR studies were conducted in high-resource settings, limiting the extent of its evaluation of equity and access in low-resource environments. In addition to small sample sizes, a lack of objective skill metrics, single training sessions, high production cost, and ergonomic constraints [40], successful AR implementation depends on infrastructure requirements such as reliable high-speed internet, compatible hardware, and technical support. The availability of such infrastructure across many LMIC training institutions is uncertain and inconsistently reported, complicating assessments of real-world feasibility and scalability [43]. Subsequently, well-designed studies conducted directly within low-resource settings are required to evaluate whether AR-based training can be equitably and practically implemented. 

Research Gaps, Limitations, and Future Directions

Overall, AR is a potential tool for surgical training, but there are gaps in the literature that need to be addressed before its implementation. Future research should employ more rigorous and standardized designs, including clearly-defined retention intervals (e.g., three to six months), validated objective performance metrics and assessment of skill transfer to real-world clinical settings. Studies conducted in low-resource settings are needed to evaluate feasibility and equity, alongside cost-effectiveness and infrastructure requirements. Such approaches would strengthen the evidence base for AR integration into surgical training.

Our scoping review has limitations. Firstly, since this is a scoping review, we did not conduct a thorough quality appraisal of each study. Secondly, publication bias may be present, as studies reporting negative or null findings are less likely to be published and therefore underrepresented. Moreover, heterogeneity in study design, interventions, and outcome measures limited direct comparisons of effectiveness. Finally, limiting inclusion to English-language publications may have introduced language bias. 

## Conclusions

This scoping review highlights the growing potential of AR in surgical education, applied across three domains: implementation into surgical curricula, skill retention and equity in low-resource settings. AR interventions were primarily focused on image overlay and visual guidance for novice learners, demonstrating short-term improvements in procedural efficiency and spatial understanding. However, evidence on long-term skill retention, transfer to real-world clinical practice, and standardized curriculum integration remains limited. Similarly, while AR has the potential to provide low-cost and decentralized training opportunities, most studies were conducted in high-resource environments, and real-world feasibility in LMICs is yet to be thoroughly evaluated. 

Future research should focus on rigorous, longitudinal, and equity-oriented studies to assess skill retention over longer intervals, objective performance metrics and transfer to real clinical environments. In addition, studies implemented directly in low-resource settings are needed to demonstrate feasibility, cost-effectiveness, and infrastructure requirements. Addressing these gaps in AR will be critical to ensure AR technology can be effectively, equitably and sustainably integrated into surgical training, overall enhancing learning outcomes and potentially improving patient care. 

## Appendices

PubMed search strategy (example)

Database: PubMed (MEDLINE) Search date: 21 August 2025 Search fields: Title/Abstract Language restrictions: English Article type restrictions: None

Concept 1: Augmented Reality Technologies

("augmented reality"[Title/Abstract] OR "mixed reality"[Title/Abstract] OR "AR"[Title/Abstract] OR "telestration"[Title/Abstract] OR "HoloLens"[Title/Abstract] OR "telementoring"[Title/Abstract])

Concept 2: Surgical Education and Procedures

("surgical training"[Title/Abstract] OR "surgical education"[Title/Abstract] OR "surgical curriculum"[Title/Abstract] OR "laparoscopic"[Title/Abstract] OR "suture"[Title/Abstract] OR "suturing"[Title/Abstract])

Concept 3: Training Outcomes, Implementation, and Access

("training"[Title/Abstract] OR "simulation"[Title/Abstract] OR "skill retention"[Title/Abstract] OR "implementation"[Title/Abstract] OR "feasibility"[Title/Abstract] OR "low-resource"[Title/Abstract] OR "LMIC"[Title/Abstract] OR "equity"[Title/Abstract] OR "access"[Title/Abstract])

Concept 4: Learner Population

("medical student"[Title/Abstract] OR "medical students"[Title/Abstract] OR "surgical trainee"[Title/Abstract] OR "surgical trainees"[Title/Abstract] OR "resident"[Title/Abstract] OR "residents"[Title/Abstract] OR "doctor"[Title/Abstract] OR "physician"[Title/Abstract])

Exclusion Criterion

NOT ("virtual reality"[Title/Abstract])

*Final Combined Search Strategy* ( ("augmented reality"[Title/Abstract] OR "mixed reality"[Title/Abstract] OR "AR"[Title/Abstract] OR "telestration"[Title/Abstract] OR "HoloLens"[Title/Abstract] OR "telementoring"[Title/Abstract]) ) AND ( ("surgical training"[Title/Abstract] OR "surgical education"[Title/Abstract] OR "surgical curriculum"[Title/Abstract] OR "laparoscopic"[Title/Abstract] OR "suture"[Title/Abstract] OR "suturing"[Title/Abstract]) ) AND ( ("training"[Title/Abstract] OR "simulation"[Title/Abstract] OR "skill retention"[Title/Abstract] OR "implementation"[Title/Abstract] OR "feasibility"[Title/Abstract] OR "low-resource"[Title/Abstract] OR "LMIC"[Title/Abstract] OR "equity"[Title/Abstract] OR "access"[Title/Abstract]) ) AND ( ("medical student"[Title/Abstract] OR "medical students"[Title/Abstract] OR "surgical trainee"[Title/Abstract] OR "surgical trainees"[Title/Abstract] OR "resident"[Title/Abstract] OR "residents"[Title/Abstract] OR "doctor"[Title/Abstract] OR "physician"[Title/Abstract]) ) NOT ("virtual reality"[Title/Abstract])
 

## References

[1] Yan Y, Krusing MB, Awad MM, Stefanidis D (2025). Challenges and opportunities to advance surgical education: a qualitative study. Global Surg Educ.

[2] Heskin L, Simms C, Holland J, Traynor O, Galvin R (2019). A systematic review of the educational effectiveness of simulation used in open surgery. Simul Healthc.

[3] Kovoor JG, Gupta AK, Gladman MA (2021). Validity and effectiveness of augmented reality in surgical education: a systematic review. Surgery.

[4] Milgram P, Takemura H, Utsumi A, Kishino F (1994). Augmented reality: a class of displays on the reality-virtuality continuum. Telemanipul Telepresence Technol.

[5] Puladi B, Ooms M, Bellgardt M (2022). Augmented reality-based surgery on the human cadaver using a new generation of optical head-mounted displays: development and feasibility study. JMIR Serious Games.

[6] Tricco AC, Lillie E, Zarin W (2018). PRISMA extension for scoping reviews (PRISMA-ScR): checklist and explanation. Ann Intern Med.

[7] Vera AM, Russo M, Mohsin A, Tsuda S (2014). Augmented reality telementoring (ART) platform: a randomized controlled trial to assess the efficacy of a new surgical education technology. Surg Endosc.

[8] Heinrich F, Huettl F, Schmidt G, Paschold M, Kneist W, Huber T, Hansen C (2021). HoloPointer: a virtual augmented reality pointer for laparoscopic surgery training. Int J Comput Assist Radiol Surg.

[9] Ghenbot Y, Ahmad HS, Chauhan D (2024). Effects of augmented reality on thoracolumbar pedicle screw instrumentation across different levels of surgical experience. World Neurosurg.

[10] Acar A, Atoum J, Reed A, Li Y, Kavoussi N, Wu JY (2024). Intraoperative gaze guidance with mixed reality. Healthc Technol Lett.

[11] Kambakamba P, Naiem A, Betz E (2024). Applying augmented reality in teaching of surgical residents-telementoring, a "stress-free" way to surgical autonomy?. Langenbecks Arch Surg.

[12] Lovett M, Ahanonu E, Molzahn A, Biffar D, Hamilton A (2024). Optimizing individual wound closure practice using augmented reality: a randomized controlled study. Cureus.

[13] Yeo CT, Ungi T, U-Thainual P, Lasso A, McGraw RC, Fichtinger G (2011). The effect of augmented reality training on percutaneous needle placement in spinal facet joint injections. IEEE Trans Biomed Eng.

[14] Felinska EA, Fuchs TE, Kogkas A (2023). Telestration with augmented reality improves surgical performance through gaze guidance. Surg Endosc.

[15] Nagayo Y, Saito T, Oyama H (2022). Augmented reality self-training system for suturing in open surgery: a randomized controlled trial. Int J Surg.

[16] Guerrini F, Bertolino L, Safa A (2024). The use of technology-based simulation among medical students as a global innovative solution for training. Brain Sci.

[17] Cizmic A, Müller F, Wise PA (2023). Telestration with augmented reality improves the performance of the first ten ex vivo porcine laparoscopic cholecystectomies: a randomized controlled study. Surg Endosc.

[18] Feifer A, Delisle J, Anidjar M (2008). Hybrid augmented reality simulator: preliminary construct validation of laparoscopic smoothness in a urology residency program. J Urol.

[19] Logishetty K, Western L, Morgan R, Iranpour F, Cobb JP, Auvinet E (2019). Can an augmented reality headset improve accuracy of acetabular cup orientation in simulated THA? A randomized trial. Clin Orthop Relat Res.

[20] Alaraj A, Charbel FT, Birk D (2013). Role of cranial and spinal virtual and augmented reality simulation using immersive touch modules in neurosurgical training. Neurosurgery.

[21] Wu L, Seibold M, Cavalcanti NA (2025). A novel augmented reality-based simulator for enhancing orthopedic surgical training. Comput Biol Med.

[22] Berger L, Civilla L, Dodier P, Rössler K, Moscato F (2025). Smartwatch-based wrist tremor assessment in neurosurgical simulator training. Sci Rep.

[23] Coelho G, Figueiredo EG, Rabelo NN, Rodrigues de Souza M, Fagundes CF, Teixeira MJ, Zanon N (2020). Development and evaluation of pediatric mixed-reality model for neuroendoscopic surgical training. World Neurosurg.

[24] Alonso-Silverio GA, Pérez-Escamirosa F, Bruno-Sanchez R, Ortiz-Simon JL, Muñoz-Guerrero R, Minor-Martinez A, Alarcón-Paredes A (2018). Development of a laparoscopic box trainer based on open source hardware and artificial intelligence for objective assessment of surgical psychomotor skills. Surg Innov.

[25] Moglia A, Marsilio L, Rossi M (2024). Mixed reality and artificial intelligence: a holistic approach to multimodal visualization and extended interaction in knee osteotomy. IEEE J Transl Eng Health Med.

[26] Hadida Barzilai D, Tejman-Yarden S, Yogev D (2025). Augmented reality-guided mastoidectomy simulation: a randomized controlled trial assessing surgical proficiency. Laryngoscope.

[27] Wolf K, Bowyer M, Bradley M, Franklin B, Weissbrod E, Dinnen R, Andreatta P (2024). Clinical readiness: can providers learn to perform lower leg fasciotomy through a tablet-based augmented reality surgical training environment?. Mil Med.

[28] Shaaban A, Tos SM, Mantziaris G, Rios-Zermeno J, Almeida JP, Quinones-Hinojosa A, Sheehan JP (2024). Assessment of high-fidelity anatomical models for performing pterional approach: a practical clinic in American Association of Neurological Surgeons Meeting 2024. World Neurosurg.

[29] Cuba M, Vanluchene H, Murek M (2024). Training performance assessment for intracranial aneurysm clipping surgery using a patient-specific mixed-reality simulator: a learning curve study. Oper Neurosurg.

[30] Ropelato S, Menozzi M, Michel D, Siegrist M (2020). Augmented reality microsurgery: a tool for training micromanipulations in ophthalmic surgery using augmented reality. Simul Healthc.

[31] Jain PP, Banerjee P, Mandal S (2024). Transforming training and education of minimally invasive surgeries using augmented reality enabled workflow. IEEE Int Conf Teach Assess Learn Eng (TALE).

[32] Shepard L, Im C, Li O, Schuler N, Holler T, Saxton A, Ghazi A (2025). Comparison of remote mixed reality versus in-person training of ultrasound-guided percutaneous nephrolithotomy with urological residents. Urology.

[33] Öztürk S, Aydoseli A, Andıç E, Şahin MS, Kaplan T, Ündeğer Ç (2025). Impact of augmented reality-based neuronavigation on neurosurgical education and training. J Istanb Fac Med.

[34] Nugent E, Shirilla N, Hafeez A, O'Riordain DS, Traynor O, Harrison AM, Neary P (2013). Development and evaluation of a simulator-based laparoscopic training program for surgical novices. Surg Endosc.

[35] Stone NN, Wilson MP, Griffith SH (2022). Remote surgical education using synthetic models combined with an augmented reality headset. Surg Open Sci.

[36] Colman S, El-Bahnasawi M, Abdulkader N, Aloul Z, Brown J, Luthra P, Rawaf D (2025). The ‘LapAR’ augmented reality training device in surgical simulation: a multi-center pilot study. Global Surg Educ.

[37] Kahol K, Ashby A, Smith M, Ferrara JJ (2010). Quantitative evaluation of retention of surgical skills learned in simulation. J Surg Educ.

[38] Abosheisha M, Prabhu R, Abdelglil M (2025). The role of augmented reality in surgical training: a narrative review. Cureus.

[39] Zhang R, Jin X, Liu M, Tong HY (2025). The effectiveness of augmented reality/mixed reality in medical education: a meta-analysis. BMC Med Educ.

[40] Abu Halimah J, Mojiri ME, Ali AA (2024). Assessing the impact of augmented reality on surgical skills training for medical students: a systematic review. Cureus.

[41] Suresh D, Aydin A, James S, Ahmed K, Dasgupta P (2023). The role of augmented reality in surgical training: a systematic review. Surg Innov.

[42] Arboleda V, Lajevardi A, Barletti P, Medina M, Ramanujam A, Elsouri KN, Demory M (2024). Augmented reality (AR) in surgery in low- and middle-income countries (LMICs): a scoping review. Cureus.

[43] Anyinkeng AB, Girma SM, Maurice T, JohnPaul E, Hiwot T, Awad AK (2025). The role of remote and virtual surgical training in expanding cardiothoracic surgical capacity in low-resource regions. BMC Surg.

